# Improving Fingerprint Verification Using Minutiae Triplets

**DOI:** 10.3390/s120303418

**Published:** 2012-03-08

**Authors:** Miguel Angel Medina-Pérez, Milton García-Borroto, Andres Eduardo Gutierrez-Rodríguez, Leopoldo Altamirano-Robles

**Affiliations:** 1 Centro de Bioplantas, Universidad de Ciego deÁvila, Carretera a Morón km 9, Ciego deÁvila ,C.P. 69450, Cuba; 2 Instituto Nacional de Astrofísica,Óptica y Electrónica. Luis Enrique Erro No. 1, Sta. María Tonanzintla, Puebla, C.P. 72840, México

**Keywords:** fingerprint verification, minutiae descriptor, minutiae triplet

## Abstract

Improving fingerprint matching algorithms is an active and important research area in fingerprint recognition. Algorithms based on minutia triplets, an important matcher family, present some drawbacks that impact their accuracy, such as dependency to the order of minutiae in the feature, insensitivity to the reflection of minutiae triplets, and insensitivity to the directions of the minutiae relative to the sides of the triangle. To alleviate these drawbacks, we introduce in this paper a novel fingerprint matching algorithm, named M3gl. This algorithm contains three components: a new feature representation containing clockwise-arranged minutiae without a central minutia, a new similarity measure that shifts the triplets to find the best minutiae correspondence, and a global matching procedure that selects the alignment by maximizing the amount of global matching minutiae. To make M3gl faster, it includes some optimizations to discard non-matching minutia triplets without comparing the whole representation. In comparison with six verification algorithms, M3gl achieves the highest accuracy in the lowest matching time, using FVC2002 and FVC2004 databases.

## 1. Introduction

Fingerprints are the marks made by ridges and furrows in the fingers. They are formed during the sixth month of intrauterine life of human beings and do not disappear until some time after death. Since there are not two persons with the same fingerprints and they do not change naturally, they are an important element to identify people.

Due to the high complexity of fingerprint matching and the huge amount of existing fingerprints, it is necessary to build computer systems that automatically process fingerprints with high accuracy and low computational costs. Historically, fingerprint recognition systems were mostly used in forensic sciences, but the current popularity of these systems is mainly due to civilian applications such as the control of physical access to facilities, the control of logical access to software, and the control of voters during elections. A key component in fingerprint recognition systems is the fingerprint matching algorithm.

To measure the quality of a fingerprint matching algorithm, the evaluator must consider the context in which it is applied. For example, the algorithms based on microscopic characteristics are suitable for applications where fingerprints are acquired with high resolution sensors (1,000 dpi or higher); the quality of these algorithms dramatically decreases when the resolution is low [1]. The following quality parameters are proved to be important for evaluating general matchers:

- Low computational cost: The algorithm satisfies memory and time restrictions for its application context [2].
- Invariance to translation: The algorithm returns a high similarity value when comparing fingerprints from the same finger notwithstanding that fingerprints be translated horizontally and/or vertically [3].
- Invariance to rotation: The algorithm returns a high similarity value when comparing fingerprints from the same finger in spite of fingerprints rotation [3].
- Tolerance to non-linear distortion: The algorithm returns a high similarity value when comparing fingerprints from the same finger even when fingerprints are affected by non-linear distortion, as a result of fingerprint creation mechanisms [4].
- Sensitivity to the individuality of fingerprints: The algorithm returns a high similarity value when comparing fingerprints from the same finger and returns a low similarity value when comparing fingerprints from different fingers [5].
- Insensitivity to select a single alignment: The algorithm does not perform a single global alignment from the best local alignment. Maximizing the local similarity value does not guarantee to find a true matching local structure pair. Even if the selected local structure pair is a true matching pair, it is not necessarily the best pair to carry out fingerprint alignment [3].
- Tolerance to partial fingerprints: The algorithm returns a high similarity value when comparing fingerprints from the same finger even when the fingerprints are not complete [5]. Partial fingerprints can be produced by the restrictions of the sensors, latent fingerprints in crime scenes, and different fingerprint creation mechanisms.
- Tolerance to the low quality of fingerprints: The results of the algorithm are not significantly affected by low fingerprint quality [6]. Due to different skin conditions and/or the different fingerprint creation mechanisms, sometimes many details of the fingerprints do not appear clearly.
- Tolerance to errors of the feature extractor: The algorithm returns a high similarity value when comparing fingerprints from the same finger even when the feature extractor has missed some features and/or has extracted some non-existent features [3].
- Determinism: Two executions of the algorithm with the same parameters return the same results.

Modern technologies impose new challenges to fingerprint matching algorithms; new systems reside on light architectures, need standards for systems interoperability, and use small area sensors [3,4,7]. In these contexts, the algorithms with higher quality are those based on minutiae [2]. Minutiae are the points where the ridge continuity breaks and they are typically represented as (*x, y, θ*); where (*x, y*) represent the 2D point coordinates, and *θ* the ridge direction at that point.

As a minutia-based matcher should be invariant to translation and rotation, the process of minutia pairing is ambiguous [8]. Thus, most matchers in this family use local minutia structures (*minutiae descriptors*) to quickly establish the minutiae correspondences [4].

A simple and accurate minutiae descriptor is based on minutiae triplets [3]. Minutiae triplets are local structures represented by three minutiae. Algorithms based on minutiae triplets have the following advantages, which make them of higher quality than algorithms based on other representations:

- They are tolerant to fingerprint deformations [9].
- They are faster and more accurate, compared to algorithms based on other representations [10,11], especially in applications with partial fingerprints [12].
- They are suitable for applications based on interoperability standards because the most popular standards are based only on minutiae [2].
- They are appropriate for systems embedded on light architectures because the representation and comparison of minutiae triplets can be performed efficiently [13].
- Minutiae triplets have higher discriminative power than minutiae pairs and single minutiae [10].

**Figure 1.**
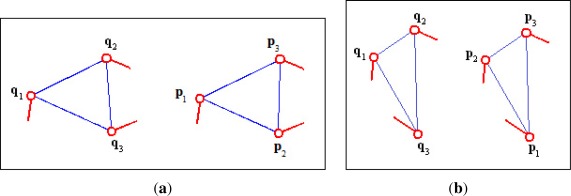
Similar minutiae triplets that were not classified as true matching by some algorithms because in image (**a**) the features are arranged according to the length of the sides, in image (**b**) the algorithms try to match the main minutia **q_1_** (left triplet) with the main minutia **p_1_** (right triplet).

**Figure 2.**
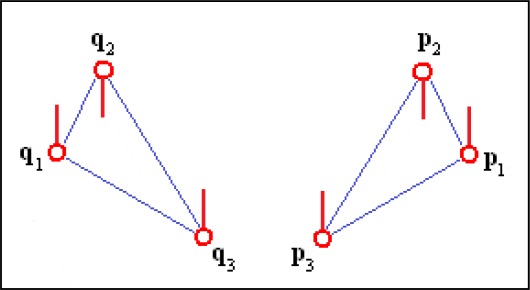
Minutiae triplets that do not match because (**p_1_, p_2_, p_3_**) is a reflected version of (**q_1_, q_2_, q_3_**).

**Figure 3.**
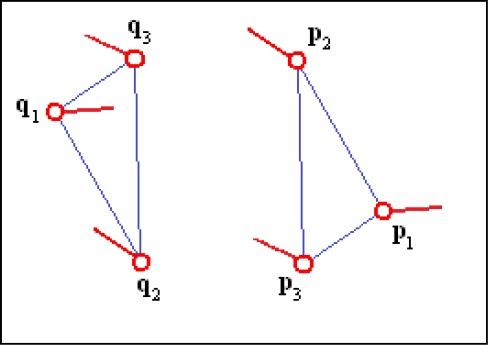
Minutiae triplets that do not match because minutiae pairs (**q_1_, p_1_**), (**q_2_, p_2_**) and (**q_3_, p_3_**) highly differ in the directions of the minutiae relative to the sides of the triangles.

Some important quality parameters related to fingerprint matching algorithms based on minutiae triplets are:

- Invariance to the order of minutiae in the feature: No matter the minutiae order in the triplet, the algorithm finds the correct correspondences of minutiae when matching similar triplets (Figure 1).
- Sensitivity to the reflection of minutiae triplets: The algorithm does not match a triplet with its reflected version (Figure 2).
- Sensitivity to the directions of the minutiae relative to the sides of the triangle: In order to find similar triplets, the algorithm takes into account the directions of the minutiae relative to the sides of the triangles formed by the triplets (Figure 3).

State-of-the-art algorithms based on minutiae triplets do not fulfil all the quality parameters, which has a negative impact on their accuracy. Table 1 shows the lacking quality parameter of all the reviewed matchers, according to the following parameters:

- Invariance to the order of minutiae in the feature.
- Sensitivity to the reflection of minutiae triplets.
- Sensitivity to the directions of the minutiae relative to the sides of the triangle.
- Insensitivity to select a single alignment.
- Tolerance to errors of the feature extractor.
- Determinism.

In this research, we introduce M3gl, a new fingerprint matcher that fulfils most quality parameters. M3gl is based on a new representation and a new comparison function for minutia triplets. An experimental comparison with algorithms based on different types of minutiae descriptors shows that M3gl is highly accurate and it has acceptable computational costs.

## 2. Definitions

This section defines some basic functions that we use throughout the paper. Given two minutiae **p_i_** = (*x_i_, y_i_, θ_i_*) and **p_j_** = (*x_j_, y_j_, θ_j_*), ed(**p_i_**, **p_j_**) represents the Euclidean distance between the coordinates of **p_i_** and **p_j_** Equation (1).

(1) $$\text{ed}(p_{i},p_{j})=\sqrt{{\left(x_{i}-x_{j}\right)}^{2}+{\left(y_{i}-y_{j}\right)}^{2}}$$

For two given angles *α* and *β*, ad*_π_*(*α, β*) computes the minimum angle required to superpose two vectors with the same origin and angles *α* and *β* respectively using Equation (2), while ad_2_*_π_*(*α, β*) computes the angle required to rotate a vector with angle *β* in clockwise sense to superpose it to another vector with the same origin and angle *α* using Equation (3).

(2) $${\text{ad}}_{\pi }(\alpha ,\beta )=\text{min}(|\alpha -\beta |,2\pi -|\alpha -\beta |)$$

(3) $${\text{ad}}_{2\pi }(\alpha ,\beta )=\{\begin{array}{ll} \beta -\alpha & \text{if}\;\beta >\alpha \\ \beta -\alpha +2\pi & \text{otherwise} \end{array}$$

Finally, for a given pair of minutiae **p_i_** and **p_j_**, ang(**p_i_, p_j_**) computes the angle of the vector with initial point at **p_i_** and terminal point at **p_j_** using Equation (4).

(4) $$\text{ang}(p_{i},p_{j})=\{\begin{array}{ll} \text{arctan}(\Delta y/\Delta x) & \text{if}\;\Delta x>0\wedge \Delta y\geq 0\\ \text{arctan}(\Delta y/\Delta x)+2\pi & \text{if}\;\Delta x>0\wedge \Delta y<0\\ \text{arctan}(\Delta y/\Delta x)+\pi & \text{if}\;\Delta x<0\\ \pi /2 & \text{if}\;\Delta x=0\wedge \Delta y>0\\ 3\pi /2 & \text{if}\;\Delta x=0\wedge \Delta y<0 \end{array}$$

where Δ*y* = *y_i_* − *y_j_* and Δ*x* = *x_i_* − *x_j_*.

## 3. Feature Representation

**Figure 4.**
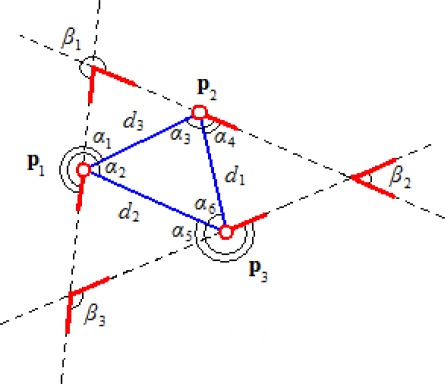
The components of the new feature representation proposed in this paper.

In this section, we introduce *m-triplets*, a robust feature representation based on minutiae triplets. Provided that a fingerprint is described by the minutia set *P*, our representation is a tuple with the following components (see Figure 4):

- minutiae **p_i_** *∈ P* are clockwise arranged starting on **p_1_**.
- *d*_*i*∈1...3_, where *d_i_* is the Euclidean distance between the minutiae different than **p_i_**.
- *d_max_*, *d_mid_* and *d_min_* are the maximum, middle and minimum distances in the triplet, respectively. Although these components can be calculated based on distance values, we store them because they are a key point for the algorithm optimizations.
- *α*_*i*∈1...6_ are the angles ad_2_*_π_*(ang(**p, q**),*θ*) required to rotate the direction *θ* of a minutia to superpose it to the vectors associated with the other two minutiae in the triplet.
- *β_i_* = ad_2*π*_ (*θ_j_, θ_k_*) is the angle required to rotate the direction of the minutia **p_k_** in order to superpose it to the direction of the minutia **p_j_**.

The m-triplets are sensitive to the directions of the minutiae relative to the sides of the triangle (angles *α*). The minutiae in this representation are arranged in clockwise direction without a central minutia. Therefore, in order to compare m-triplets, the similarity function considers the three minutiae rotations in clockwise sense (next section contains a formal definition of this procedure). This representation and the comparison function guarantee the invariance to the order of minutiae in the feature, and the sensitivity to the reflection of minutiae triplets.

## 4. M-Triplets Similarity

In this section, we introduce a new m-triplets similarity. It is designed to accurately distinguish between similar and non-similar m-triplets. Given two m-triplets **t** and **r**, we propose s_inv_(**t**, **r**) in Equation (5) to compare m-triplets.

(5) $$s_{\text{inv}}(t,r)=\text{max}\left\{s_{\text{part}}(t,r),s_{\text{part}}\left(t,\mathrm{shift}(r)\right),s_{\text{part}}\left(t,\mathrm{shift}(\mathrm{shift}(r))\right)\right\}$$

where: *shift*(**r**) is the clockwise-shifted m-triplet **r** and s_part_(**t, r**) is the base similarity function in Equation (6).

(6) $$s_{\text{part}}(t,r)=\{\begin{array}{ll} 0\;\;\text{if}\;s_{\theta }(t,r)=0\vee s_{d}(t,r)=0\vee s_{\alpha }(t,r)=0\vee s_{\beta }(t,r)=0 & \\ 1-(1-s_{d}(t,r))(1-s_{\alpha }(t,r))(1-s_{\beta }(t,r)), & \text{otherwise} \end{array}$$

The base similarity function s_part_ is defined using functions s*_θ_*, s_d_, s*_α_*, and s*_β_*, which consider different components of the m-triplets. According to Equation (6), two m-triplets are totally dissimilar if they have at least one component totally dissimilar. If all component similarities are above zero, the product rule makes the similarity hight if at least one component is close to 1.

The invariance to rotation is an important quality parameter for fingerprint recognition, especially for fingerprint identification. Nevertheless, for fingerprint verification, the rotation is usually restricted by sensors. The function s*_θ_* Equation (7) incorporates this information into the m-triplets similarity to increase minutia discrimination for such problems. Consequently, two m-triplets are dissimilar if their minutiae directions differ more than *π*/4.

(7) $$s_{\theta }(t,r)=\{\begin{array}{ll} 0 & \text{if}\;\exists i({\text{ad}}_{\pi }(\theta_{i}^{t},\theta_{i}^{r})>\pi /4)\\ 1 & \text{otherwise} \end{array}$$

The function s_d_ Equation (8) compares m-triplets in terms of the side lengths of the triangle formed by the triplet minutiae. s_d_ returns values in the interval [0,1], returning 0 if at least one length difference is greater than threshold *t_l_*. s_d_ returns 1 if all length differences are 0; that is, the triangles formed by both m-triplets are identical.

(8) $$s_{d}(t,r)=\{\begin{array}{ll} 0 & \text{if}\;\exists i(|d_{i}^{t}-d_{i}^{r}|\;>\;t_{l})\\ 1-{\text{max}}_{i=1...3}\left\{|d_{i}^{t}-d_{i}^{r}|\right\}/t_{l} & \text{otherwise} \end{array}$$

The function s*_α_* Equation (9) compares m-triplets based on the angles formed by minutiae directions and the sides of the triangles (angles *α* on Figure 4).

(9) $$s_{\alpha }(t,r)=\{\begin{array}{ll} 0 & \text{if}\;\exists i({\text{ad}}_{\pi }(\alpha_{i}^{t},\alpha_{i}^{r})\;>\;t_{\alpha })\\ 1-{\text{max}}_{i=1...6}\left\{{\text{ad}}_{\pi }(\alpha_{i}^{t},\alpha_{i}^{r})\right\}/t_{\alpha } & \text{otherwise} \end{array}$$

The function s*_β_* Equation (10) compares m-triplets based on relative minutiae directions (angles *β* on Figure 4

(10) $$s_{\beta }(t,r)=\{\begin{array}{ll} 0 & \text{if}\;\exists i({\text{ad}}_{\pi }(\beta_{i}^{t},\beta_{i}^{r})\;>\;t_{\alpha })\\ 1-{\text{max}}_{i=1...3}\left\{{\text{ad}}_{\pi }\left(\beta_{i}^{t},\beta_{i}^{r}\right)\right\}/t_{\alpha } & \text{otherwise} \end{array}$$

Equations (9) and (10) return values in the interval [0, 1]. They return 0 if at least two compared angles differ more than threshold *t_a_*. The less the angles differ, the higher is the value returned by the equations; therefore, they return 1 if the compared angles are identical.

In order to detect dissimilar m-triplets in advance, avoiding all costly shifts, we use Theorems 1, 2, and 3. Proofs for Theorems 1 and 2 appear in the appendixes. Proof of Theorem 3 is similar to the proof of Theorem 1 and is not stated.

**Theorem 1.** *Given two m-triplets* **t** *and* **r**, *if* $\left|d_{\max}^{t}-d_{\max}^{r}\right|>t_{l}$ *then* s_inv_(**t, r**) = 0.

**Theorem 2.** *Given two m-triplets* **t** *and* **r***, if* $\left|d_{\mathrm{mid}}^{t}-d_{\mathrm{mid}}^{r}\right|>t_{l}$ *then* s_inv_(**t, r**) = 0.

**Theorem 3.** *Given two m-triplets* **t** *and* **r***, if* $\left|d_{\min}^{t}-d_{\min}^{r}\right|>t_{l}$ *then* s_inv_(**t, r**) = 0.

Based on these theorems, we can modify s_inv_ Equation (5). This way, our m-triplets similarity can be re-written as Equation (11).

(11) $$s_{\text{inv}}(t,r)=\{\begin{array}{l} 0\;\text{if}(|d_{\max}^{t}-d_{\max}^{r}|\;>t_{l})\vee (|d_{\mathrm{mid}}^{t}-d_{\mathrm{mid}}^{r}|\;>t_{l})\vee (|d_{\min}^{t}-d_{\min}^{r}|\;>t_{l})\\ \text{max}\{s_{\text{part}}(t,r),s_{\text{part}}(t,\mathrm{shift}(r)),s_{\text{part}}(t,\mathrm{shift}(\mathrm{shift}(r)))\},\;\;\text{otherwise} \end{array}$$

Local distance threshold *t_l_* and angle threshold *t_a_* are parameters of the algorithm and must be tuned according to the image characteristics.

The function s_inv_, by means of s_part_, achieves the invariance to translation and the invariance to restricted rotation, the tolerance to non-linear distortion and the sensitivity to the directions of the minutiae relative to the sides of the triangle. The clockwise shifting makes function s_inv_ invariant to the order of minutiae in the feature and sensitive to the reflection of minutiae triplets.

## 5. M3GL Algorithm

In this section, we introduce M3gl, a new fingerprint verification algorithm based on the proposed minutiae triplet representation and similarity measure.

Given a fingerprint described by the minutia set *P* we compute the m-triplets as follows. For each **p** ∈ *P*, find its *c* nearest minutiae in *P* and build all m-triplets that include **p** and two of its nearest minutiae, discarding duplicates. This way of computing m-triplets makes M3gl tolerant to the low quality of fingerprints and tolerant to errors of the feature extractor. Additionally, m-triplets in the fingerprint are sorted according to the length of the largest side to perform a binary search when looking for similar m-triplets; Theorem 1 guarantees the safety of this procedure.

M3gl consists of the following major steps: local minutiae matching, global minutiae matching, and similarity score computation.

### 5.1. Local Minutiae Matching

This step finds the similar m-triplets in the template fingerprint using binary search. Then, it sorts all matching pairs according to the similarity value and finds the local matching minutiae. Formally, the algorithm is the following:

1. Let *Q* and *P* be the query and template fingerprint minutiae sets respectively. Let *R* and *T* be the query and template fingerprint m-triplets sets respectively. Let *A* ← {} be the set that will contain local matching m-triplets pairs.
2. For each query m-triplet **r_i_** ∈ *R* perform binary search looking for the template m-triplets {**t_1_, t_2_**, …, **t_u_**} ⊂ *T* with similarity value higher than 0 and add the pairs (**r_i_, t_1_**), (**r_i_, t_2_**), …, (**r_i_, t_u_**) to *A*.
3. Sort in descendant order all matching pairs (**r, t**) in *A* according to the similarity value.
4. Let *M* ← {} be the set containing local matching minutiae pairs.
5. For each (**r**, **t**) ∈ *A* do
  - Let *B ←* {(**q_1_, p_1_**), (**q_2_, p_2_**), (**q_3_, p_3_**) } be the matching minutiae that maximizes s_inv_(**r, t**) where **q_1_, q_2_, q_3_** *∈ Q* and **p_1_, p_2_, p_3_** *∈ P.*
  - For each (**q_i_, p_i_**) *∈ B* do:
    - If there is not any pair (**q_j_, p_j_**) *∈ M* that **q_j_** = **q_i_** or **p_j_** = **p_i_** then *M ← M ∪* {(**q_i_, p_i_**)}.

### 5.2. Global Minutiae Matching

This step uses every minutiae pair as a reference pair for fingerprint rotation and performs a query minutiae transformation for each reference pair. Later, it selects the transformation that maximizes the amount of matching minutiae. This strategy overcomes the limitations of the single alignment based matching. M3gl uses three criteria to determine if two minutiae match at global level and to achieve the tolerance to non-linear distortion. First, the Euclidean distance must not exceed threshold *t_g_*. Second, minutia directions must not exceed threshold *t_a_*. Third, the directions differences relative to reference minutiae pair must not exceed threshold *t_a_*. The formal algorithm is the following:

1. Let *n ←* 0 be the maximum number of matching minutiae computed after performing all fingerprint rotations.
2. For each (**q_i_, p_i_**) ∈ *M* do:
  - Let *E ←* {} be the set containing global matching minutiae pairs for the current iteration.
  - For each (**q_j_, p_j_**) *∈ M*, if *∀*(**q_k_, p_k_**) *∈ E* [(**q_j_** *≠* **q_k_**) *∧* (**p_j_** *≠* **p_k_**)]
    - Compute **q***^′^* = (*x^′^, y^′^, θ^′^*) as $\left[\begin{array}{c} x^{\prime }\\ y^{\prime }\\ \theta^{\prime } \end{array}\right]=\left[\begin{array}{ccc} \cos(\Delta \theta ) & -\sin(\Delta \theta ) & 0\\ \sin(\Delta \theta ) & \cos(\Delta \theta ) & 0\\ 0 & 0 & 1 \end{array}\right]\;\;\left[\begin{array}{c} x_{3}-x_{1}\\ y_{3}-y_{1}\\ \theta_{3}-\theta_{1} \end{array}\right]+\left[\begin{array}{c} x_{2}\\ y_{2}\\ \theta_{2} \end{array}\right]$ where **q_i_** = (*x*_1_, *y*_1_, *θ*_1_), **p_i_** = (*x*_2_, *y*_2_, *θ*_2_), Δ*θ* = *θ*_2_ *− θ*_1_, **q_j_** = (*x*_3_, *y*_3_, *θ*_3_).
    - Let **p_j_** = (*x*_4_, *y*_4_, *θ*_4_); if ed(**q**^′^, **p_j_**) ≤ *t_g_* ∧ ad_π_ (ad_2π_(*θ*_2_, *θ*_1_), ad_2π_(*θ*_4_, *θ*_3_)) *≤ t_a_* ∧ ad*_π_*(*θ^′^*, *θ*_4_) *≤ t_a_*, then *E ← E ∪* {(**q_j_, p_j_**)}.
  - if *n < |E|* then *n ← |E|*.

### 5.3. Similarity Score Computation

The similarity value is computed using the formula $\frac{n^{2}}{\left|P||Q\right|}$; where *P* and *Q* are the template and query fingerprint minutiae sets respectively, and *n* is the amount of matching minutiae pairs.

Global distance threshold *t_g_* and angle threshold *t_a_* are parameters of the algorithm, and must be tuned based on the image characteristics.

## 6. Experimental Results

In order to evaluate the new fingerprint matching algorithm, we use databases DB1_A, DB2_A, DB3_A and DB4_A of FVC2002 and FVC2004 competitions. These databases are commonly used as benchmarks for evaluating fingerprint matchers in the context of fingerprint verification. We also use the FVC evaluation protocol [22]. Performance is measured by indicators EER, FMR100, FMR1000 and ZeroFMR. The indicator Time refers to the average matching time in milliseconds. We carry out all the experiments on a laptop with an Intel Core i7 740QM processor (1.73 GHz) and 4 GB of RAM.

We use the same parameters values for M3gl in all databases (*t_l_* = 12, *t_g_* = 12, *t_a_* = π/6, *c* = 4). We estimate these parameters through a few experiments using fingerprint databases DB1_B, DB2_B, DB3_B and DB4_B of FVC2004 competition.

In the experimental comparisons, we include the algorithm proposed by Jiang and Yau [9] (JY) because it is the most popular algorithm based on minutiae triplets in the literature. We implement the algorithm proposed by Parziale and Niel [11] (PN) because it is the algorithm based on minutiae triplets more similar to our proposal and we are interested in showing the difference in accuracy and speed. We also compare our proposal with algorithms based on other types of minutiae descriptors, that is why we implement the algorithms proposed by Wang *et al*. [23] (WLC), Qi *et al*. [24] (QYW), Tico and Kuosmanen [25] (TK), and Udupa *et al*. [26] (UGS).

**Figure 5.**
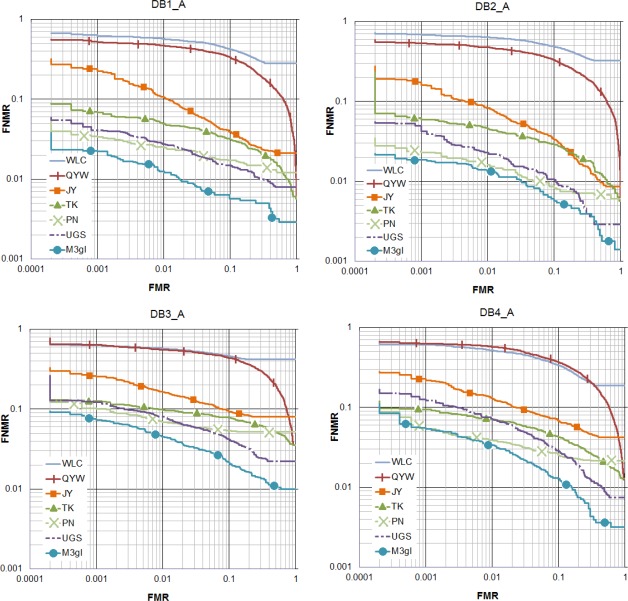
ROC curves with the performance of the compared algorithms in FVC2002.

**Figure 6.**
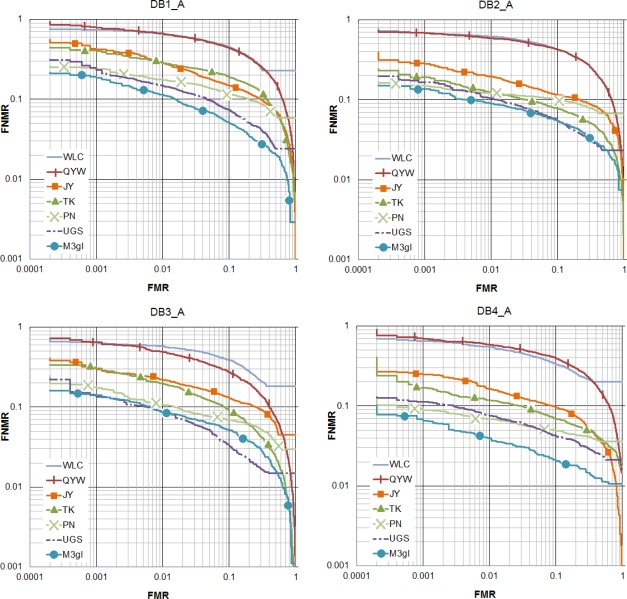
ROC curves with the performance of the compared algorithms in FVC2004.

**Figure 7.**
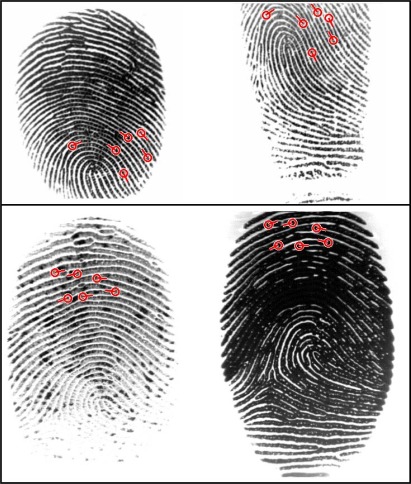
Two examples where all matching algorithms fail but our algorithm finds true matching minutiae. The first row contains fingerprints db1_36_1 and db1_36_4 of database DB1_A (FVC2002); the second row contains fingerprints 85_6 and 85_8 of database DB1_A (FVC2004).

Figures 5 and 6 show that M3gl achieves lower FNMR for most of the FMR values. Tables 2 and 3 show that our algorithm achieves the best results for most of the performance indicators. Figure 7 shows examples where our algorithm is able to find true matching minutiae in difficult cases (partial fingerprints with low overlapping, non-linear distortion and low quality) where the other algorithms fail. M3gl is also the fastest algorithm due to the following reasons:

- The m-triplets similarity function includes the properties demonstrated in Theorems 1, 2 and 3 to discard comparisons without performing all its operations.
- The algorithm performs binary search when looking for similar m-triplets in the local minutiae matching step.

The similarity score computation of M3gl is simple but robust. In order to test this robustness we substitute the similarity score computation of M3gl for the strategies proposed by Wang *et al*. [23], Qi *et al*. [24], Jiang and Yau [9], and Tico and Kuosmanen [25]; we name the new algorithms M3gl1, M3gl2, M3gl3 and M3gl4 respectively. We test M3gl and its variations in the testing databases DB1_B, DB2_B, DB3_B and DB4_B of FVC2002 and FVC2004. The experimental results (Tables 4 and 5) do not show a clear superiority of any algorithm; nevertheless, when comparing M3gl with the other algorithms by pairs, we find that M3gl wins in most of the accuracy indicators.

Experimental results confirm that fulfilling the quality parameters discussed in Section 1 has a direct impact in the matcher accuracy.

## 7. Conclusions

Fingerprint matching algorithms based on minutiae triplets have proved to be fast and accurate. They are commonly used on light architectures and in systems based on interoperability standards for fingerprints represented by minutiae. However, existing algorithms have several limitations that affect their accuracy. In this paper, we identify the quality parameters that have a more significant impact on fingerprint matching accuracy in order to create M3gl, a more accurate matcher.

M3gl uses a new representation based on minutiae triplets and a new comparison function that achieves the invariance to the translation and rotation of fingerprints, and achieves the sensitivity to the directions of the minutiae relative to the sides of the triangle. The new representation arranges the minutiae in clockwise sense and the comparison function performs the three possible rotations of the triplets achieving the invariance to the order of minutiae in the feature and the sensitivity to the reflection of minutiae triplets. The components of the proposed representation are compared using thresholds that allow the tolerance to the non-linear distortion of fingerprints. Besides, the new comparison function includes optimizations that avoid comparing all the components of the triplets, increasing the matching speed in many cases.

M3gl is deterministic, insensitive to select a single alignment, and sensitive to the individuality of fingerprints. It uses a simple and effective procedure to compute minutiae triplets which makes the algorithm tolerant to the low quality of fingerprints and to errors of the feature extractor.

Experimental results in databases of the FVC2002 and FVC2004 competitions show that M3gl has low computational costs and is more accurate than other algorithms based on minutiae triplets and other algorithms based on different representations.

In the near future we plan to study the behaviour of the new algorithm for latent fingerprint identification.

## Figures and Tables

**Table 1.** Summary of the lacking quality parameter on fingerprint matching algorithms based on minutiae triplets.

**Algorithms**	**Lacking quality parameter**					
	**I**	**II**	**III**	**IV**	**V**	**VI**
JY [9]	X			X	X	
KV [14]		X	X	X		
PN [11]		X			X	
JG1 [12]	X		X		X	X
JG2 [5]	X					X
RUR [13]	X		X			
TB [15]			X			X
FFCS [16]	X				X	
CTYZ [17]	X	X				
XCF [18]	X	X	X		X	
ZGZ [19]		X	X			
HK [20]		X	X			
GSM [21]			X			X

**Table 2.** Experimental results on databases DB1_A, DB2_A, DB3_A and DB4_A of FVC2002.

**Database**	**Algorithm**	**EER**	**FMR100**	**FMR1000**	**ZeroFMR**	**Time(ms)**
	WLC	29.5	57.2	63.6	66.9	123.0
D	QYW	22.8	46.8	52.6	55.9	13.6
B	JY	5.1	10.6	23.9	31.9	3.3
1	TK	4.0	4.9	7.0	8.9	12.4
-	PN	1.9	2.5	3.4	5.8	20.3
A	UGS	2.2	2.8	4.1	5.9	1,239.4
	M3gl	**1.1**	**1.3**	**2.3**	**3.8**	**1.4**

	WLC	34.0	63.6	69.3	72.6	269.6
D	QYW	22.8	47.8	53.7	59.2	27.2
B	JY	4.5	8.3	17.3	27.6	4.6
2	TK	3.6	4.6	6.3	23.1	19.1
-	PN	1.4	1.6	2.4	3.5	44.4
A	UGS	1.9	2.3	4.9	5.6	2,846.1
	M3gl	**1.3**	**1.4**	**1.9**	**2.2**	**1.6**

	WLC	29.8	57.4	62.7	65.0	27.1
D	QYW	30.0	55.7	63.5	77.9	6.3
B	JY	9.4	16.4	26.1	33.1	1.5
3	TK	7.7	9.8	12.6	16.4	5.8
-	PN	5.6	6.9	10.2	12.8	5.6
A	UGS	5.3	8.0	12.2	26.4	96.0
	M3gl	**3.1**	**4.5**	**7.5**	**9.4**	**0.6**

	WLC	22.9	51.7	61.0	63.5	37.0
D	QYW	24.3	57.3	63.2	67.6	8.5
B	JY	7.4	13.0	23.0	28.3	2.1
4	TK	5.1	7.1	9.4	12.1	8.4
-	PN	3.1	3.9	5.6	10.3	10.3
A	UGS	4.2	7.1	12.6	16.8	463.0
	M3gl	**2.4**	**3.4**	**5.6**	**11.2**	**1.0**

**Table 3.** Experimental results on databases DB1_A, DB2_A, DB3_A and DB4_A of FVC2004.

**Database**	**Algorithm**	**EER**	**FMR100**	**FMR1000**	**ZeroFMR**	**Time(ms)**
	WLC	27.3	64.8	73.9	77.3	150.0
D	QYW	24.3	60.6	80.3	97.3	15.5
B	JY	13.5	28.5	42.8	55.9	4.2
1	TK	15.9	29.1	41.8	51.0	11.7
-	PN	11.4	17.7	24.4	25.9	20.9
A	UGS	7.9	14.8	24.9	31.3	1,649.4
	M3gl	**6.3**	**11.4**	**19.3**	**21.7**	**1.3**

	WLC	28.1	62.1	68.9	78.0	103.0
D	QYW	24.8	52.1	58.9	73.7	12.9
B	JY	11.0	19.4	28.4	39.2	3.0
2	TK	7.8	12.0	18.7	24.9	11.6
-	PN	10.0	12.1	15.1	16.9	14.9
A	UGS	6.4	10.5	16.7	19.9	1,210.8
	M3gl	**6.2**	**9.1**	**13.6**	**15.3**	**1.1**

	WLC	24.5	57.9	64.9	69.5	312.0
D	QYW	19.7	47.7	65.9	87.5	22.6
B	JY	12.0	22.1	32.3	41.4	6.4
3	TK	9.6	19.7	32.9	37.3	17.0
-	PN	7.1	10.7	17.6	24.9	38.0
A	UGS	**5.1**	**8.6**	**14.1**	22.4	6,510.9
	M3gl	**6.1**	**8.6**	14.4	**16.4**	**1.9**

	WLC	23.6	54.9	65.0	70.0	108.8
D	QYW	25.5	55.5	65.8	74.7	13.3
B	JY	9.7	16.3	25.3	28.6	3.2
4	TK	7.6	11.4	16.6	39.4	11.8
-	PN	5.2	6.9	9.3	11.9	17.2
A	UGS	5.1	7.6	11.2	13.1	1,687.1
	M3gl	**3.0**	**4.0**	**6.9**	**10.3**	**1.2**

**Table 4.** Experimental results on databases DB1_B, DB2_B, DB3_B and DB4_B of FVC2002.

**Database**	**Algorithm**	**EER**	**FMR100**	**FMR1000**	**ZeroFMR**	**Time(ms)**
D	M3gl1	1.7	2.5	3.6	3.6	
B	M3gl2	2.5	**1.6**	**3.2**	**3.2**	
1	M3gl3	**1.4**	2.9	3.6	3.6	
-	M3gl4	1.6	2.9	**3.2**	**3.2**	
B	M3gl	2.0	2.5	**3.2**	**3.2**	

D	M3gl1	**0.7**	1.1	**1.8**	**1.8**	
B	M3gl2	1.9	2.5	3.2	3.2	
2	M3gl3	0.8	**0.8**	**1.8**	**1.8**	
-	M3gl4	1.9	2.9	2.9	2.9	
B	M3gl	**0.7**	1.1	**1.8**	**1.8**	

D	M3gl1	6.5	9.4	12.1	12.1	
B	M3gl2	7.7	13.9	18.6	18.6	
3	M3gl3	6.9	**9.3**	11.8	11.8	
-	M3gl4	6.9	11.4	27.9	27.9	
B	M3gl	**6.4**	**9.3**	**11.1**	**11.1**	

D	M3gl1	2.3	3.6	19.6	19.6	
B	M3gl2	2.8	3.9	**11.1**	**11.1**	
4	M3gl3	**2.2**	**3.2**	16.4	16.4	
-	M3gl4	4.0	32.5	78.2	78.2	
B	M3gl	2.4	3.6	19.6	19.6	

**Table 5.** Experimental results on databases DB1_B, DB2_B, DB3_B and DB4_B of FVC2004.

**Database**	**Algorithm**	**EER**	**FMR100**	**FMR1000**	**ZeroFMR**	**Time(ms)**
D	M3gl1	4.0	5.4	10.4	10.4	
B	M3gl2	5.8	24.3	30.7	30.7	
1	M3gl3	3.8	6.1	13.6	13.6	
-	M3gl4	6.4	33.6	50.4	50.4	
B	M3gl	**2.5**	**5**	**9.3**	**9.3**	

D	M3gl1	9.7	21.1	24.3	24.3	
B	M3gl2	12.9	25.0	28.6	28.6	
2	M3gl3	**8.9**	**18.9**	**20.7**	**20.7**	
-	M3gl4	11.4	25.0	31.1	31.1	
B	M3gl	10.5	20.7	25.7	25.7	

D	M3gl1	0.9	1.4	2.5	2.5	
B	M3gl2	3.1	6.1	24.6	24.6	
3	M3gl3	1.0	1.4	2.5	2.5	
-	M3gl4	3.7	48.2	70.4	70.4	
B	M3gl	**0.6**	**0.7**	**1.4**	**1.4**	

D	M3gl1	3.4	**6.1**	**7.5**	**7.5**	
B	M3gl2	4.6	11.4	13.2	13.2	
4	M3gl3	**3.8**	**6.1**	7.9	7.9	
-	M3gl4	5.3	20.0	72.9	72.9	
B	M3gl	4.2	**6.1**	**7.5**	**7.5**	

## Appendix Theorem Proofs

**Theorem A1.** *Given two m-triplets* **t** *and* **r***,* $\mathrm{if}\left|d_{\max}^{t}-d_{\max}^{r}\right|>t_{l}$ *then* s_inv_(**t**, **r**) = 0.

*Proof.* From the definition of m-triplet we have:

(A1) $$d_{\max}^{r}\geq d_{\mathrm{mid}}^{r}$$

(A2) $$d_{\max}^{r}\geq d_{\min}^{r}$$

(A3) $$d_{\mathrm{mid}}^{r}\geq d_{\min}^{r}$$

Assume, without loss of generality, that $d_{\max}^{t}>d_{\max}^{r}$; then, from the hypothesis of Theorem A1, we infer that:

(A4) $$d_{\max}^{t}-d_{\max}^{r}>t_{l}$$

which is equivalent to:

(A5) $$d_{\max}^{t}-t_{l}>d_{\max}^{r}$$

From Equations (A5) and (A1) we have $d_{\max}^{t}-t_{l}>d_{\mathrm{mid}}^{r}$; which is equivalent to:

(A6) $$d_{\max}^{t}-d_{\mathrm{mid}}^{r}>t_{l}$$

Similarly, from Equations (A5) and (A2) we obtain:

(A7) $$d_{\max}^{t}-d_{\min}^{r}>t_{l}$$

From Equations (A4), (A6) and (A7) we conclude that $d_{\max}^{t}$ differs more than threshold *t_l_* with respect to $d_{\max}^{r}$, $d_{\mathrm{mid}}^{r}$ and $d_{\min}^{r}$ respectively. Therefore, s_d_(**t**, **r**) returns 0 for every clockwise shifting of **r** and consequently s_inv_(**t, r**) = 0.

**Theorem A2.** *Given two m-triplets* **t** *and* **r**, $\mathrm{if}\left|d_{\mathrm{mid}}^{t}-d_{\mathrm{mid}}^{r}\right|>t_{l}$ *then* s_inv_(**t**, **r**) = 0.

*Proof.* From the definition of m-triplet we have:

(A8) $$d_{\max}^{t}\geq d_{\mathrm{mid}}^{t}$$

Assume, without loss of generality, that $d_{\mathrm{mid}}^{t}>d_{\mathrm{mid}}^{r}$; then, from the hypothesis of Theorem A2, we infer that:

(A9) $$d_{\mathrm{mid}}^{t}-d_{\mathrm{mid}}^{r}>t_{l}$$

which is equivalent to:

(A10) $$d_{\mathrm{mid}}^{t}-t_{l}>d_{\mathrm{mid}}^{r}$$

From Equation (A10) and (A3) we have $d_{\mathrm{mid}}^{t}-t_{l}>d_{\min}^{r}$; which is equivalent to:

(A11) $$d_{\mathrm{mid}}^{t}-d_{\min}^{r}>t_{l}$$

Similarly, from Equations (A8) and (A9) we obtain:

(A12) $$d_{\max}^{t}-d_{\mathrm{mid}}^{r}>t_{l}$$

Additionally, from Equations (A8) and (A11) we obtain:

(A13) $$d_{\max}^{t}-d_{\min}^{r}>t_{l}$$

From Equations (A9), (A11), (A12) and (A13) we conclude that $d_{\max}^{t}$ and $d_{\mathrm{mid}}^{t}$ differ more than threshold *t_l_* with respect to both $d_{\mathrm{mid}}^{r}$, $d_{\min}^{r}$. Therefore, s_d_(**t, r**) returns 0 for every clockwise shifting of **r** and consequently s_inv_(**t, r**) = 0.
